# Locus Number Estimation of *MHC* Class II *B* in Stone Flounder and Japanese Flounder

**DOI:** 10.3390/ijms16036000

**Published:** 2015-03-13

**Authors:** Jiajun Jiang, Chunmei Li, Quanqi Zhang, Xubo Wang

**Affiliations:** Key Laboratory of Marine Genetics and Breeding (Ocean University of China), Ministry of Education, Qingdao 266003, China; E-Mails: j15964239284@163.com (J.J.); shelleylcm212@gmail.com (C.L.); qzhang@ouc.edu.cn (Q.Z.)

**Keywords:** major histocompatibility complex, MHC, class II *B*, stone flounder, Japanese flounder, locus number

## Abstract

Members of major histocompatibility complex (MHC) family are important in immune systems. Great efforts have been made to reveal their complicated gene structures. But many existing studies focus on partial sequences of *MHC* genes. In this study, by gene cloning and sequencing, we identified cDNA sequences and DNA sequences of the *MHC* class II *B* in two flatfishes, stone flounder (*Kareius bicoloratus*) and homozygous diploid Japanese flounder (*Paralichthys olivaceus*). Eleven cDNA sequences were acquired from eight stone flounder individuals, and most of the polymorphic sites distributed in exons 2 and 3. Twenty-eight alleles were identified from the DNA fragments in these eight individuals. It could be deduced from their Bayesian inference phylogenetic tree that at least four loci of *MHC* class II *B* exist in stone flounder. The detailed whole-length DNA sequences in one individual were analyzed, revealing that the intron length varied among different loci. Four different cDNA sequences were identified from one homozygous diploid Japanese flounder individual, implying the existence of at least four loci. Comparison of the cDNA sequences to the DNA sequence confirmed that six exons existed in this gene of Japanese flounder, which was a common feature shared by Pleuronectiformes fishes. Our results proved the multi-locus feature of *MHC* class II *B*. The sequences we obtained would provide detailed and systematic data for further research.

## 1. Introduction

Major histocompatibility complex (MHC) is a series of cell surface molecules encoded by a large gene family present in all vertebrates. They occupy an important place in immune systems. Generally, MHC can be divided into three classes. Class I molecules are peptide-binding proteins that select short peptides for antigen presentation and molecules aiding antigen procession. Class II molecules include peptide-binding proteins and some proteins assisting antigen loading onto MHC class II’s peptide-binding proteins. Functioning very differently from the other two classes, Class III molecules include several secreted proteins with immune functions, such as components of the complement system, cytokines and heat shock proteins. Because of their important immune roles in all vertebrates, MHC molecules arouse great enthusiasm in researchers to explore their function mechanism in antigen processing and presentation, mate selection, transplant rejection, and their evolutionary diversity. *MHC* genes sparkle for their polymorphism, which can be embodied in their sequence variation and multi-locus. Some *MHC* loci are extraordinarily polymorphic while some are not. Researchers have drawn the first complete sequence and gene map of the human *MHC* [1]. Two hundred twenty-four identified gene loci spanned on chromosome 6, and one hundred twenty-eight of them are predicted to be expressed. These results have uncovered the extraordinary polymorphism and evolution of this region. However, there are some species whose *MHC* loci are generally monomorphic. The whale *MHC* genes, for instance, are less polymorphic than those of human [2]. In any case, the polymorphic *MHC* genes are considered to be related to disease resistance of vertebrates, assisting an organism to fight various parasites and pathogens, which partially infects the organism evolution and its living environment.

Great efforts have been made to reveal the complicate structures of teleost *MHC* genes. For example in half-smooth tongue sole (*Cynoglossus semilaevis*), two *MHC* class II *A* genes are identified [3]. And from the allele distribution in several individuals, it is inferred that at least two loci exist in each gene. By analyzing bacterial artificial chromosome (BAC) clones, researchers find nine class II *A* and 15 class II *B* loci in tilapiine fish *Oreochromis niloticus* [4]. By hybridization with specific probes and gene sequencing, two class II *A* loci and six class II *B* loci have been identified in zebrafish (*Brachydanio rerio*) [5]. In recent years, more studies have focused on the origin and evolution of genes. Researchers have demonstrated a novel class II DE group in some teleost species, and that the teleost fish class II genes can be classified into three major groups [6]. Medaka (*Oryzias latipes*) has five pairs of expressed class II genes, each comprising one II *A* and one II *B* gene, and the tightly linked II *A* and one II *B* genes have undergone conserved evolution [7]. In these studies, the accurate information of *MHC* structure and locus distribution in the genome enabled a deeper understanding of their evolution.

Despite the abundant research on *MHC* gene structure, some problems remain to be solved. Lack of clear loci delimitations and lack of integrate sequence information increase difficulties of disclosing the complexity of MHC system. Some conclusions may cause inaccurate results and misunderstanding if they are deduced from partial *MHC* sequences with ambiguous locus information. So it is necessary to distinguish *MHC* gene sequences of different loci. Here we analyzed genome sequences of stone flounder (*Kareius bicoloratus*, Basilewsky, 1855) and Japanese flounder (*Paralichthys olivaceus*, Temminck and Schlegel, 1846) *MHC* class II *B* gene, a gene encoding the β chain of MHC class II molecules. The two fish species belong to different families of Pleuronectiformes. The sequences we used were obtained by gene cloning and sequencing and some of them covered all exons and introns of the gene. In this way, we were expecting to speculate the locus number of this gene in stone flounder. Furthermore, we also identified the *MHC* class II *B* gene in Japanese flounder, and tried to estimate its locus number from a homozygous diploid genome. Enriching basic *MHC* sequence information, our study might contribute to more accurate prediction of gene locus number and assist further research on *MHC* genes.

## 2. Results

### 2.1. cDNA Polymorphism in Eight Stone Flounder Individuals

**Figure 1:**
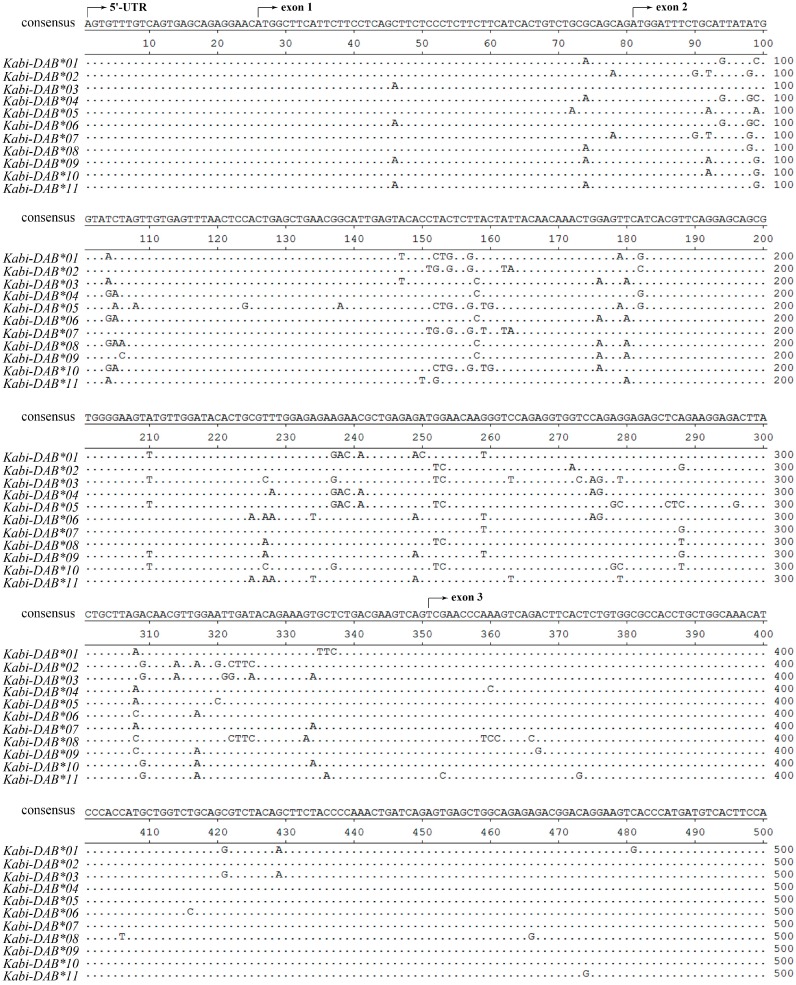

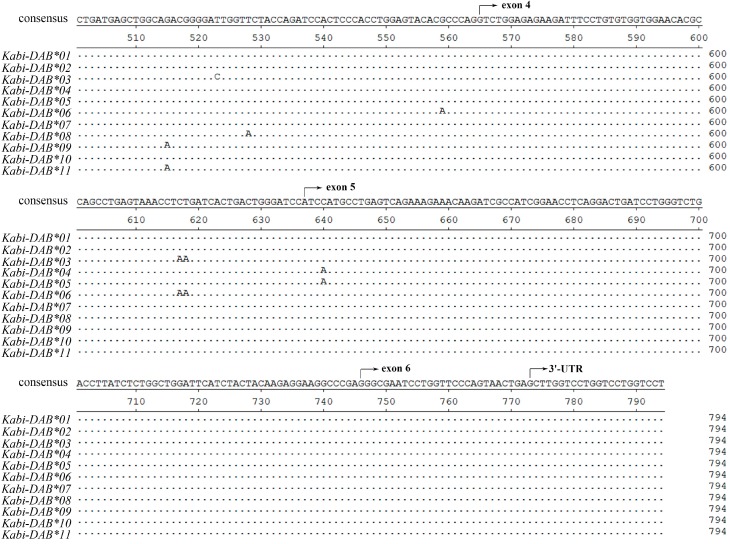
Sequence alignment of major histocompatibility complex (*MHC*) class II *B* cDNAs in eight stone flounder individuals. Nucleotides identical to the consensus are indicated by dots. The beginning of each exon is marked above the sequences.

cDNA sequences of stone flounder *MHC* class II *B* contained a 5'-UTR (untranslated region) of 25 nt, an ORF (open reading frame) of 747 nt and a 3'-UTR of 22 nt. Altogether 11 different cDNA sequences were found in eight stone flounder individuals (GenBank Nos. JX645176–JX645186). Each of these sequences was sequenced from more than three clones. Ninety-three polymorphic sites were found and such polymorphic sites mainly distributed in exons 2 and 3, while the other exons were relatively conserved (Figure 1). Analyzed by codon-based test of positive selection using the Nei-Gojobori method (*p* = 0.05), *d*_N_ (the number of non-synonymous substitutions per site) was higher than *d*_S_ (the number of synonymous substitutions per site) in peptide binding region (PBR) (*d*_N_/*d*_S_ = 2.872, *p* = 0.002), which contained exons 2 and 3. In the remained exons, *d*_N_ was close to *d*_S_ (*d*_N_/*d*_S_ = 1.161, *p* = 0.124). None of the tested individuals possessed all of the cDNA sequences. The number of sequences found in each individual ranged from one to three. Kabi-DAB*04 was found in individuals Nos. 3 and 4. Kabi-DAB*06 was found in individuals Nos. 6 and 8. Kabi-DAB*08 was carried by individuals Nos. 3, 7 and 8. Each of the other eight sequences was found only in one individual.

### 2.2. Partial DNA Sequences in Eight Stone Flounder Individuals

**Figure 2:**
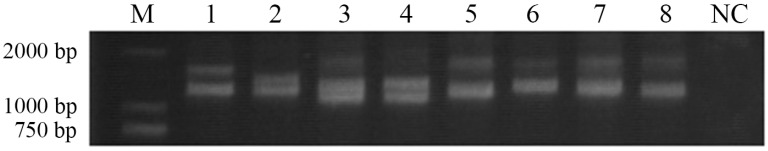
Electrophoresis patterns of *MHC* class II *B* partial DNA sequences in eight stone flounder individuals. Samples from eight individuals are labeled by numbers. NC is the PCR blank control without any template. More than one fragment between 1000 and 2000 bp was acquired from each of the eight DNA samples. The three fragments (2000, 1000 and 750 bp) in the marker (M) are marked on the left.

**Figure 3:**
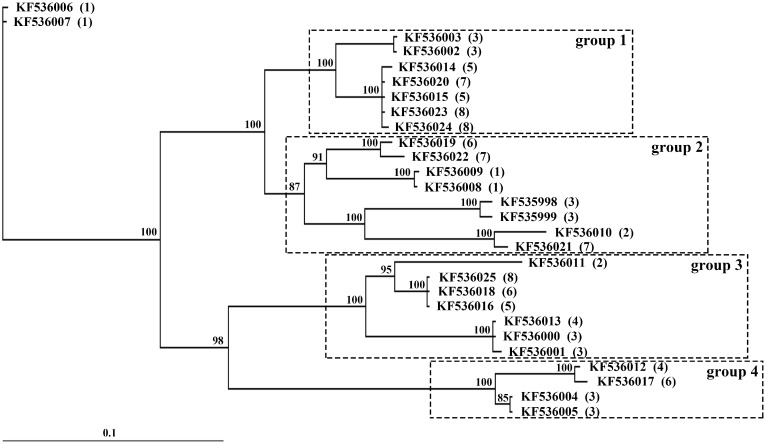
Bayesian inference phylogenetic tree of *MHC* class II *B* alleles from eight stone flounder individuals. Sequences are indicated by their Genbank numbers. The individual they are sequenced from are indicated by numbers in parentheses. The sequences are divided into four groups, boxed by dashed lines.

A pair of primers was designed to amplify exons 1 to 4 of the stone flounder *MHC* class II *B*. Fragments of different lengths were acquired from each individual, all of which were sequenced and proved to be the desired gene. The lengths of these fragments spread between 1000 and 2000 bp. Some of them could be easily distinguished by electrophoresis patterns (Figure 2). Their lengths were different due to the disparity in introns 1, 2 and 3. Altogether 28 alleles were identified. The allele number we got from each individual varied from two to eight. A Bayesian inference phylogenetic tree was constructed with all these alleles (Figure 3). At least four groups could be divided according to the position of individual No. 3 alleles. In each group, there were two alleles from individual No. 3, meaning they were likely to be a pair of alleles from the same locus, and all the alleles in the same group might be multiple alleles. KF536002, for instance, tended to be the allele form the same locus with KF536003, instead of KF535998, whose relationship was farther. We could not deny the bare possibility that KF536002 and KF 536003 were from two closely related loci. Besides, no more than two alleles of the same individual were found in the same cluster, which hindered us to further divide the groups. So it seemed that alleles from the same group might generate from at least one locus. Thus at least four loci of stone flounder gene *B* could be deduced in this tree. KF536006 and KF536007 were not included for the lack of more close related sequences. And from the sequenced clones, we did not find any group shared by all the individuals.

### 2.3. Whole-Length DNA Sequences in One Stone Flounder Individual

Though the results from eight individuals gave a general impression of *MHC* class II *B* loci, the exact situation remained unclear. Would there be any intron length disparity in the other introns? What might be the reasons of different intron lengths? Besides, when studying heterozygous diploids, alleles from too many individuals might generate confused sequences and distraction. So in the following procedures, only individual No. 3, which seemed to carry more loci in the previous study, was chosen and its whole-length DNA sequences of *MHC* class II *B* were analyzed.

**Figure 4:**
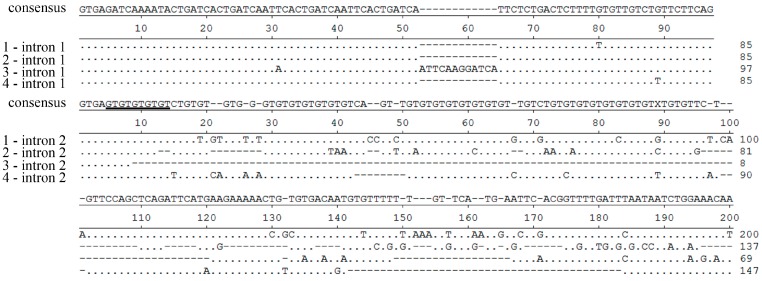

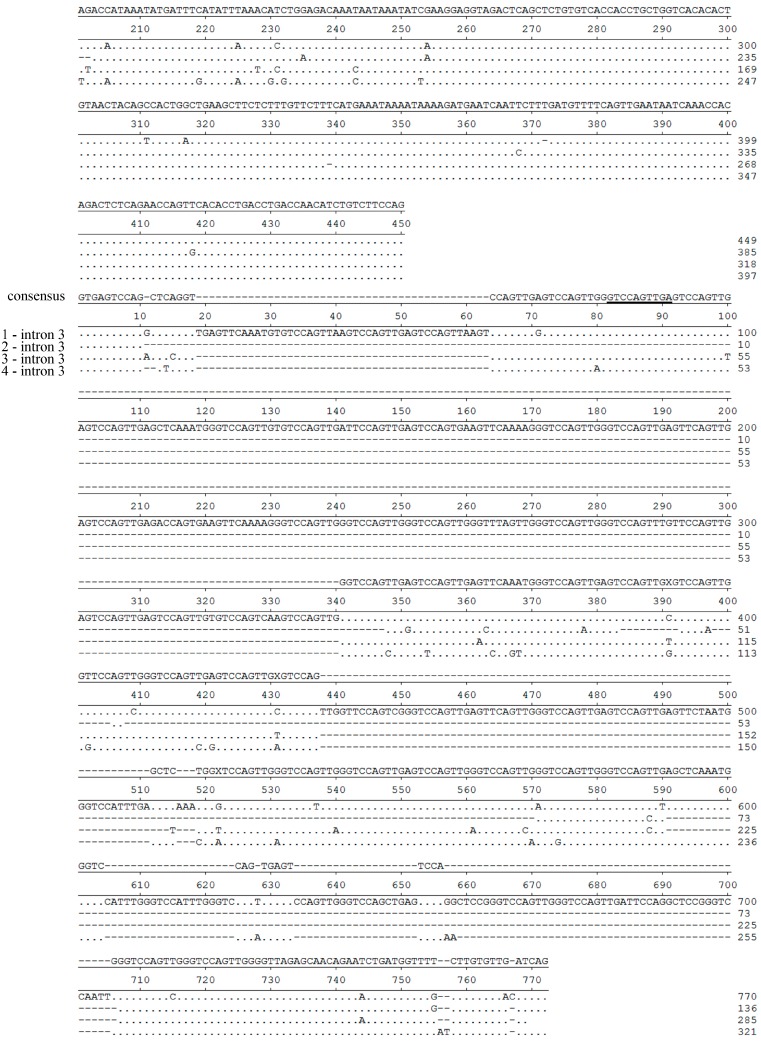

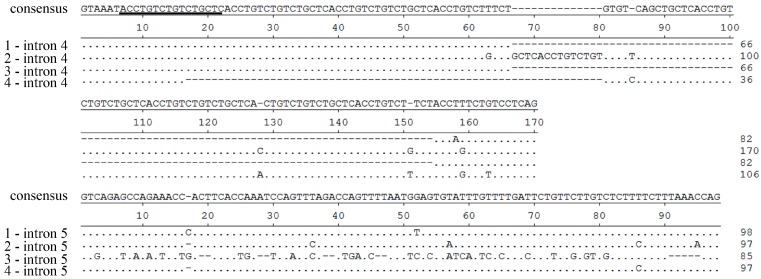
Intron sequences alignment of *MHC* class II *B* presumed locus 1–4 in stone flounder individual No. 3. The identical nucleotides are indicated by dots and gaps are indicated by dashes. The repeating units (GT, GTCCAGTTGA, and ACCTGTCTGTCTGCTC) in intron 2, intron 3 and intron 4 are underlined.

Forty-two clones were randomly chosen to sequence for the whole-length of *MHC* class II *B*. Altogether eight alleles (Genbank Nos. KF535998–KF536005) were acquired, implying at least four loci existed in this heterozygous diploid individual. Together with the results mentioned above, these eight alleles might come from at least four presumed loci. We chose four sequences (KF535998, KF536000, KF536002 and KF536004) from each of the four groups (Figure 3) to represent alleles from four different loci (presumed locus 1 to locus 4, respectively). Lengths of introns from different loci differed greatly (Figure 4), and there were also great intron differences in the last two introns. Locus 3 had a special 12 bp insertion in intron 1. In intron 2, numbers of GT repeats contributed to the different lengths, and there were also more nucleotide substitutions in this intron than others. While in intron 3, the lengths varied greatly among loci, which were 770, 136, 285 and 321 bp, respectively. There was a decamer unit GTCCAGTTGA repeating in this region, which, to some extent, caused the different length in intron 3. The length of intron 4 was 82, 106, or 170 bp, with several repeats of a 16 bp motif ACCTGTCTGTCTGCTC. Lengths did not vary greatly in intron 5, and locus 3 contained a specific intron 5 sequence compared to the other loci.

### 2.4. Sequences in the Homozygous Diploid Japanese Flounder Individual

**Figure 5:**
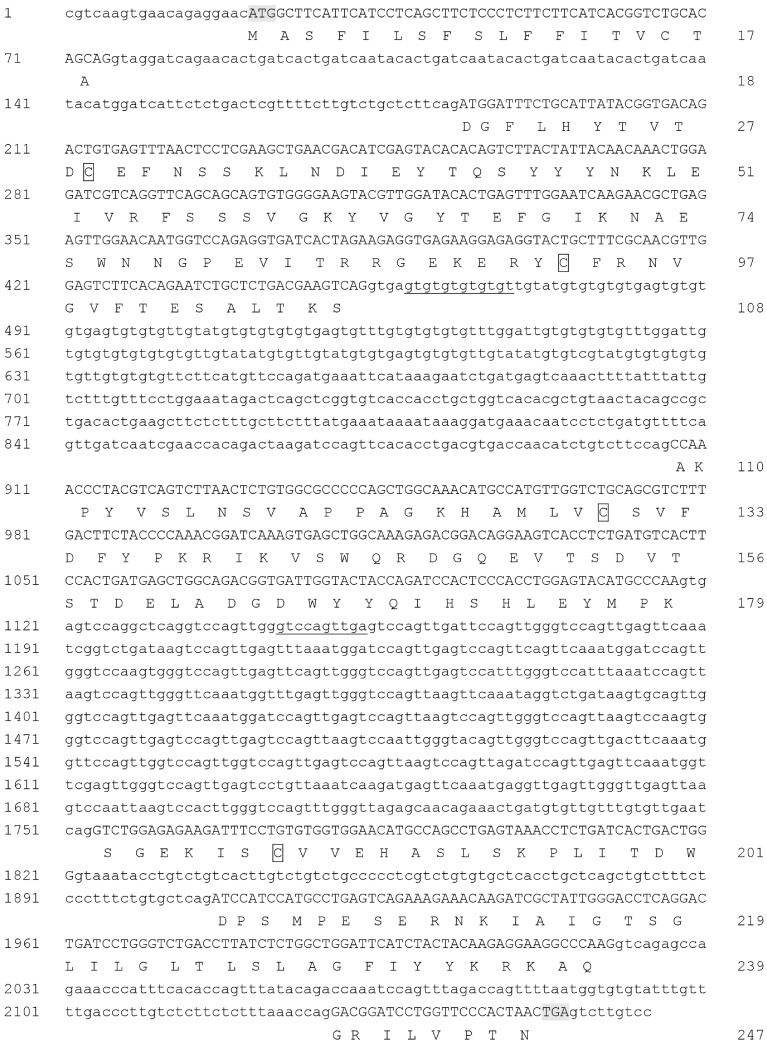
DNA sequence and putative amino acid sequence of the Japanese flounder *MHC* class II *B*. The intron sequences are in lowercase letters and the exon sequences in DNA are in uppercase letters. The amino acid sequences encoded by the open reading frame (ORF) are in uppercase letters below the exon sequences. The translational initiation codon and termination codon are in shade. Four cysteines are boxed. The start of GT repeat in intron 2 and the decamer repeat GTCCAGTTGA in intron 3 are underlined.

The 774 nt cDNA fragment of *MHC* class II *B* was amplified in the homozygous Japanese flounder individual. It contained a complete 744 nt ORF encoding 247 amino acid residues (Figure 5). The first eighteen amino acid residues encoded by exon 1 were predicted to form a signal peptide. A total of 40 clones were sequenced, and four alleles were identified based on their specific amino acid sequences, namely Paol-DAB*001–Paol-DAB*004 (Nos. KJ784490–KJ784493). Paol-DAB*001 had the highest frequency (23 among 40 clones). Sequences existing in only one or two clones were excluded from the analysis. Only five polymorphic sites were found in cDNA sequences, two of which were synonymous substitution and the other three were non-synonymous substitution. The four sequences differed from each other in three amino acid sites. Paol-DAB*003 had a synonymous substitution, 645A>T, and Paol-DAB*002 also had one, 242A>G. Compared to Paol-DAB*001, three non-synonymous substitutions occurred, namely, E74G in Paol-DAB*002, L10H and A162T in Paol-DAB*004.

The DNA sequence of Japanese flounder *MHC* class II *B* (No. KJ784489) was 2164 bp in length. It was comprised of six exons and five introns. The microsatellite repeat GT in the first part of intron 2 and the ten base pair repeat GTCCAGTTGA in intron 3 were common features shared by Japanese flounder and stone flounder.

### 2.5. Sequence Comparison among Species

**Figure 6:**
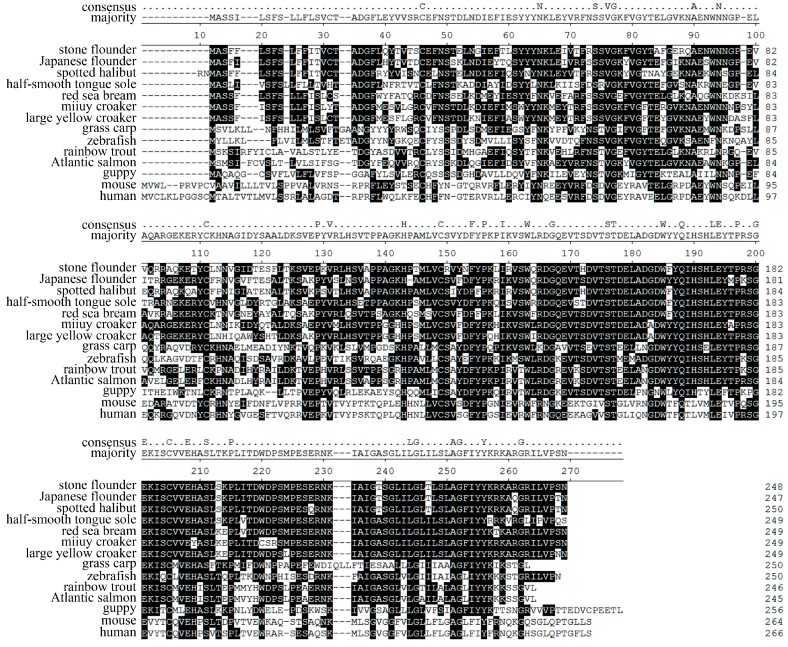
Alignment of peptides encoded by *MHC* class II *B* in fourteen species. Gaps are indicated by dashes. Conserved sites are marked black. The consensus sites are indicated on top. The Genbank numbers of these sequences are: AFY98547 (stone flounder), AII02001 (Japanese flounder), ADB43564 (spotted halibut), AAP20186 (red sea bream), ABV48909 (large yellow croaker), ADV36785 (miiuy croaker), AEM75094 (grass carp), CAD87794 (zebrafish), AAD53026 (rainbow trout), CAA49726 (Atlantic salmon), AAC34836 (guppy), P18469 (mouse), and AAA59781 (human).

**Figure 7:**
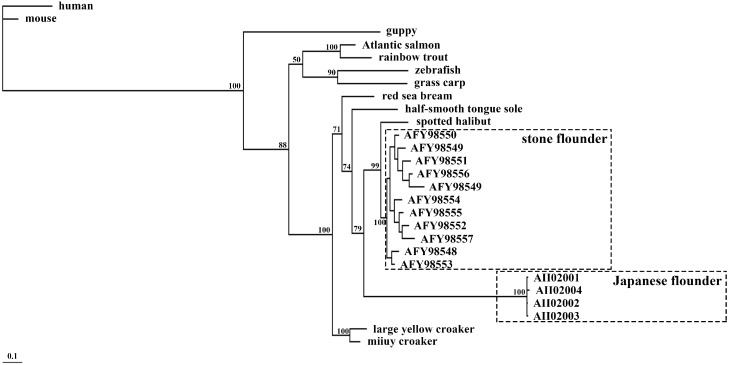
Phylogenetic tree of peptides encoded by *MHC* class II *B* in fourteen species. The human MHC class II B was set as an outgroup. The 11 alleles in stone flounder and the four alleles in Japanese flounder are indicated by their Genbank numbers. Sequences from other species are the same to those in Figure 6.

The amino acid sequences deduced from the stone flounder and the Japanese flounder *MHC* class II *B* were aligned with their orthologs. The peptide encoded by Japanese flounder *MHC* class II *B* shared 72.5%, 77.7% and 80.6% with those encoded by half-smooth tongue sole, stone flounder and spotted halibut, respectively. Four cysteine residues in the mature peptide were conserved among species (Figure 6). The first part of these peptides was variable, while the latter part was more conserved, which might help to maintain the structural integrity of the molecule. The phylogenetic tree showed that peptides encoded by the Japanese flounder and the stone flounder *MHC* class II *B* clustered together first and then clustered with their flatfish orthologs (Figure 7). Both stone flounder and spotted halibut are in family Pleuronectidae, and the peptides encoded by their *MHC* class II *B* shared identity of 80.2%.

**Figure 8:**
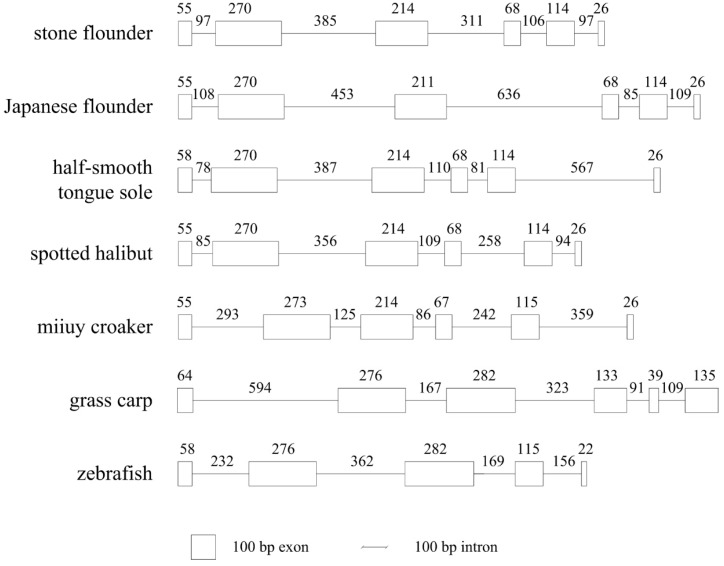
*MHC* class II *B* structures of seven fish species. Exons are indicated by boxes and introns are indicated by lines. The taxonomy status of these species is: stone flounder: Pleuronectidae; Japanese flounder: Paralichthyidae; half-smooth tongue sole: Cynoglossidae; spotted halibut: Pleuronectidae; miiuy croaker: Sciaenidae; grass carp: Cyprinidae; and zebrafish: Cyprinidae.

By comparing the DNA sequence of Japanese flounder *MHC* class II *B* with its orthologs from order Pleuronectiformes, we noticed that they all possessed six exons and five introns (Figure 8). While the intron lengths differed, the lengths of their exons were almost the same. Thus the peptides they encoded were conserved to some extent.

## 3. Discussion

Due to the lack of BAC library and whole genome information of stone flounder, here in this study, we used two strategies to minimize errors. The first strategy was about primer design. There was no sequence disparity in the 5'-UTR or 3'-UTR when conducting rapid amplification of cDNA ends (RACE) for stone flounder gene *B* [8]. So the primers for amplifying the whole-length cDNA and DNA sequences were designed on the UTR regions. When amplifying the partial DNA sequences in eight individuals, we designed the primers on the relatively conserved region of exons 1, 3 and 4, according to the cDNA sequences (Figure 1). The trial in partial gene *B* sequence amplification provided new proofs for the complication of gene *B* structure. We only used one pair of primers to amplify the whole-length DNA sequence of this gene in stone flounder or Japanese flounder. Thus all the exons and introns could be amplified once, which ensured the accuracy of gene structure analysis. Considering the conservation of UTR sequences, we could speculate the sequences got in this study were mostly comprehensive. But we could not exclude the possibility that the RACE primers were not degenerate enough to acquire all UTR sequences. Another strategy was using high fidelity DNA polymerase in every PCR system, conducting multiple batches with the same templates and primers in different thermocyclers at different time, and sequencing as many clones as possible. High fidelity DNA polymerase could reduce the proportion of amplification errors. Repeating the same experimental batches at different time could reduce the percent of wrong sequences. Meanwhile, we set a standard that each sequence used in analysis was verified by at least three clones to guarantee their accuracy. But it might also exclude sequences that were expressed lower or that were harder to be amplified. So the standard we used might be a compromise. Combining the two strategies, we supposed the sequences got in this study to be relatively comprehensive and accurate.

Many studies have focused either on limited subset of *MHC* loci or on partial sequences, such as PBR and its flanking sequences. These indistinct locus definitions may impose much redundant information on MHC studies. Some researchers have emphasized the significance of complete *MHC* class II *B* sequences covering the full genetic diversity present in a species, which can provide a useful tool to compare the molecular evolution of these genes between different groups of vertebrates [9]. In zebrafish (*Brachydanio rerio*), three possible loci have been primarily identified base on 20 cDNA *MHC* class II *B* sequences. But after investigating the intron sequences, the presence of a fourth locus is inferred [10]. In three-spined stickleback (*Gasterosteus aculeatus*), fifteen distinct class I exon 2 and exon 3 sequences are assigned to twelve loci based on their intron 2 length differences, and they are further grouped into three families derived from different ancestral genes [11]. Though results deduced from partial sequences of *MHC* may not fundamentally affect the precision of analysis, discrimination of *MHC* loci by whole-length sequences does matter in related studies. Here in stone flounder, we found some alleles with similar intron 1 sequences might also differ greatly in the other intron sequences (Figure 3). The phenomenon furnishes valid evidence that the *MHC* genome sequences may contain much more information than ever expected.

Homozygous or haploid genome is beneficial for locus number studies of *MHC* genes. With a single copy of the *MHC* class II *B* gene, potbellied seahorse (*Hippocampus abdominalis*) provides an ideal system for *MHC*-related studies [12]. For lack of such materials, the genome of a single individual offers easier access to locus number analysis of the gene, especially in heterozygous diploid materials. But the disparity of locus number among different individuals should not be ignored. In a study of cichlid *MHC* class II *B*, altogether 17 different loci have been found in various cichlid species, but probably none of these individuals carry all the loci in a single haplotype, varying from one to thirteen per individual [13]. In this study, we estimated locus number from partial DNA sequences of eight individuals, and analyzed each intron disparity from whole-length sequences of only one individual. The rough speculation showed at least four *MHC* class II *B* loci existed in this species and they were not carried by all the individuals. To practice locus number analysis in homozygous individuals, a homozygous diploid Japanese flounder was used in this study. Not surprisingly, the level of *MHC* class II *B* polymorphism was lower than that of diploid individuals in terms of its cDNA polymorphism. By cloning the whole-length DNA sequence, no fragment of different lengths was observed from the electrophoresis pattern. It could be conjectured that if more loci of this gene were found in Japanese flounder, their sequences would differ in intron lengths.

The genome structure of *MHC* class II *B* is not very conserved among different teleosts. Previous genome structure comparison has revealed the presence of an extra intron in stone flounder [8]. In this study, it was also found in Japanese flounder, which was different from the result in an earlier study [14]. This extra intron between exons 3 and 4 has been demonstrated in the Percomorpha and Atherinomorpha rather than non-acanthopterygian fishes. It arises after the divergence of Ostariophysi and Protacanthopterygii, and before the divergence of Acanthopterygii [15]. The extra intron has also been proved in grass carp (*Ctenopharyngodon idella*) [16], miiuy croaker (*Miichthys miiuy*) [17], spotted halibut (*Verasper variegatus*) [18], and half-smooth tongue sole (*Cynoglossus semilaevis*) [19], but not in zebrafish (*Brachydanio rerio*) [5]. The variant genome structures of *MHC* class II *B* may help to understand its evolution. The four fish species in Pleuronectiformes all possess this extra intron and conserved *MHC* class II *B* structures, despite the different families they belong to. Both the Perciformes fish miiuy croaker and the Cypriniformes fish grass carp have this extra intron. Another Cypriniformes species zebrafish, however, does not have it. It can be reasonably inferred that this extra intron is a common feature shared by Pleuronectiformes fishes, and the structure may vary in other orders of fish. A repeat hexamer CCAGGT is conspicuous in this extra intron of perch-like fish [15]. Noticing the 5' (GT) and 3' (AG) splice sites in this hexamer, the authors hypothesize that additional duplications of the hexamer increase once a tandem duplication locates in this region. Intriguingly, in the present study, the repeat decamer GTCCAGTTGA in stone flounder and Japanese flounder also included 5' and a 3' splice sites, which inclined to the former hypothesis.

Microsatellites have been detected in introns and flanking regions of *MHC* genes in many species. In house mice (*Mus domesticus*), a series of microsatellites in and near to *MHC* provide a simple and inexpensive method to discriminate haplotypes [20]. In human, the association between some microsatellites and haplotypes provide a powerful tool for studying genetic drift and admixture of populations [21]. So far, some species have been proved to possess locus-specific intron length variation, most of which generates from microsatellites. In a study of two species of cichlid fishes, the amplified segment lengths of *MHC* class II *B* vary from 335 to 457 bp, with different repeat times of a 12-nucleotide element in intron 1. This can serve as a marker for the classification of genes into groups [22]. In half-smooth tongue sole, two distinct loci of *MHC* class II *B* are identified by different nucleotides and lengths of intron 1, which also possess AC repeats [23]. In the stone flounder and Japanese flounder *MHC* class II *B*, rich repeats, such as GT and GTCCAGTTGA, were found distributing in introns. The difference of their repeating numbers contributed to diverse intron lengths. In the intron 4 of Japanese flounder *MHC* class II *B*, there was only one copy of ACCTGTCT, which was similar to the repeat unit in stone flounder. It was reasonable to suspect their roles in changing introns length of different gene loci in diploid or polyploidy genomes, albeit only a DNA sequence from one locus had been acquired in the homozygous Japanese flounder individual.

Pseudogenes are not rare in *MHC* family. In bovine, two out of three *DRB* genes are pseudogenes [24]. In six different class II loci of the zebrafish (*Brachydanio rerio*), three are truncated pseudogenes [5]. A class I pseudogene in the rat is distinctive for an additional stop codon in exon 2 [25]. Here in the No. 3 stone flounder individual, each of the four loci corresponded to one transcriptional product. So with the present results, no pseudogene could be proved in stone flounder *MHC* class II *B* gene. By alignment of different cDNA sequences from stone flounder, polymorphic sites were found distributing mainly in PBR. The constrained distribution has also been found in many MHC studies, which can be elucidated by the protein binding function of PBR. Some researchers have proposed that the *d*_N_/*d*_S_ pattern is different between PBR and other regions [26]. As was shown in the stone flounder *MHC* class II *B*, *d*_N_ was higher than *d*_S_ in PBR. While in the remained regions, the two figures were similar. Positive selection might play a prominent role in PBR evolution of the stone flounder *MHC* class II *B*.

The multi-locus feature of *MHC* genes is an exemplification of their highly polymorphism. Just as at least four *MHC* class II *B* were found in one stone flounder individual, the duplicated *MHC* genes might enhance the diversity of immune response in this species. And it was possible to find more loci of this gene in stone flounder. However, the more *MHC* gene loci may not mean the better immune ability. Organisms balance between a high immune ability and a moderate pressure they can stand. Too much disparate MHC molecules may occupy much space on the cytomembrane. As proposed by some researchers, increasing the number of MHC molecules per individual will increase the number of foreign peptides that can be presented and the number of different T-cell receptors positively selected in the thymus. But it will also reduce the number of TCRs by negative selection [27]. So the number of MHC molecules expressed in an individual is constrained in actual situations.

The present study provided information for the complexity of *MHC* genes structure and established a feasible method to infer the locus number of such genes. At least four loci of *MHC* class II *B* were found in eight stone flounder individuals. We also examined this gene in one heterozygous diploid individual and in a homozygous genome, and discovered its sequence features. These results would assist further extensive polymorphism studies and development of molecular markers to distinguish individuals and populations. They would also help to deduce the evolutionary relationship of *MHC* genes.

## 4. Experimental Section

### 4.1. Fish and Sampling

Eight healthy wild stone flounders (four females and four males) were caught in the Yellow Sea, northern China. They were sexually mature. The gender could be distinguished by the morphology of their gonads, and female individuals were much bigger than males. These sample fish were numbered 1 to 8. One homologous Japanese flounder individual was provided by Institute of Oceanology, Chinese Academy of Sciences [28]. Muscle tissue samples were kept in ethanol at room temperature until use. Spleen tissue samples of each fish individual were preserved at −80 °C until use. Animal experiments were performed according to the Regulations for the Administration of Affairs Concerning Experimental Animals (China, 1988). The procedures were also approved by College of Marine Life, Ocean University of China (Qingdao, China).

### 4.2. DNA and RNA Extraction and cDNA Synthesis

Genomic DNA was extracted from muscle samples of each individual with phenol-chloroform method. 0.03 g of each sample was used for DNA extraction. The extracted DNA samples were stored at −20 °C until use. Total RNA was extracted from spleen samples of each individual with Trizol reagent (Invitrogen, Carlsbad, CA, USA) according to the manuals. Then DNA was removed by DNase I (TaKaRa, Dalian, China). The extracted RNA samples were stored at −80 °C until use. The quality and quantity of extracted DNA and RNA were detected by electrophoresis and Nanophotometer Pearl (Implen GmbH, Munich, Germany). First-strand cDNA in the spleen was synthesized with PrimeScript™ RT-PCR Kit (TaKaRa) according to the manuals.

### 4.3. Primer Design and Cloning

In a previous study, the whole-length sequences of *MHC* class II *B* cDNA (GenBank Nos. GQ273943 and GQ273944) and one whole-length DNA sequence (GenBank No. JX647846) were identified in stone flounder [8]. Here, primers SF-Fw1 (forward) (5'-GAGTGTTTGTCAGTGAGCAGAGG-3') and SF-Rv1 (reverse) (5'-CAGTCCAGCAGATGGCAGC-3') were used to amplify the complete ORF of the cDNA (GenBank Nos. JX645176–JX546186). They were also for the amplification of the whole-length DNA sequence (GenBank Nos. KF535998–KF536005). SF-Fw2 (5'-CTTCTCCCTCTTCTTCATCAC-3') and SF-Rv2 (5'-TCCACCACACAGGAAATCTT-3') were used for amplification of partial DNA sequence from exon 1 to exon 4 (GenBank Nos. KF536006–KF536025). For *B* gene in Japanese flounder, primers JF-Fw3 (5'-TCGTCAAGTGAACAGAGGAAC-3') and JF-Rv3 (5'-GGACAAGACTCAGTTAGTGGGAA-3') were designed to amplify the ORF of cDNA (GenBank Nos. KJ784490–KJ784493) and the corresponding DNA sequence (GenBank No. KJ784489) based on a known sequence [14]. The PCR system was 25 μL with about 100 ng template DNA or cDNA. The reaction was catalyzed by Platinum^®^ *Taq* DNA Polymerase High Fidelity (Invitrogen, Carlsbad, CA, USA). PCR products were separated by agarose gel electrophoresis and purified with Quick Gel Extraction Kit (CWBIO, Beijing, China). Purified fragments were ligated to *pEASY*™-T1 Cloning vectors (TransGen Biotech, Beijing, China) and cloned into Trans5α Chemically Competent Cell (TransGen Biotech). For each fragment, clones were screened by PCR with M13 primers and 8 to 20 positive ones were sequenced. Same sequences acquired from more than two clones were selected for analysis. The sequences were nominated according to the nomenclature proposed in 1990 [29].

### 4.4. Sequence Alignment and Data Analysis

Sequences were processed by software EditSeq and aligned by MegAlign in Lasergene 7. Signal peptides were predicted by SignalP 4.1 Server [30]. Rates of synonymous substitution and non-synonymous substitution were calculated by MEGA 6. Repeated fragments were found by Tandem Repeats Finder [31]. The Bayesian inference phylogenetic trees were constructed by MrBayes version 3.2.2 [32], using General Time Reversible (GTR) substitution model and γ-distributed rate variation.
